# Adherence in allergen immunotherapy: Current situation and future implications 

**DOI:** 10.5414/ALX02318E

**Published:** 2022-11-21

**Authors:** Francesca Gehrt, Qingqing Xu, Ilaria Baiardini, Giorgio Walter Canonica, Oliver Pfaar

**Affiliations:** 1 Department of Otorhinolaryngology, Head and Neck Surgery, Section of Rhinology and Allergy, University Hospital Marburg, Philipps-University Marburg, Marburg, Germany,; 2 Department of Allergy, Beijing Tong Ren Hospital, Capital Medical University,; 3 Department of Otolaryngology Head and Neck Surgery, Beijing Tong Ren Hospital, Capital Medical University, Beijing, PR China,; 4 Personalized Medicine, Asthma and Allergy, Humanitas Clinical and Research Center, IRCCS, Rozzano, and; 5 Department of Biomedical Sciences, Humanitas University, Pieve Emanuele, Milan, Italy; *Denotes equal contribution

**Keywords:** allergen immunotherapy, subcutaneous, sublingual, adherence, persistence

## Abstract

Allergen immunotherapy (AIT) is the only disease-modifying treatment in allergy. However clinical trials as well as real-life studies revealed poor treatment adherence. This article is intended to provide an overview of the current literature of the last 10 years, to outline reasons for poor treatment adherence in AIT and to provide possible solutions for improving adherence.

## Introduction 

Allergic rhino-conjunctivitis affects ~ 30% of the world population, and studies indicate that prevalence rates are increasing worldwide [1]. 

To date, allergen immunotherapy (AIT) is the only disease-modifying therapeutic option available and has been applied for over a century since its introduction by Leonard Noon [2, 3, 4]. Even though the mechanisms of action behind AIT have not been fully understood yet, the sustained effect, the efficacy, and safety of AIT have been demonstrated in various randomized controlled trials (RCTs) as well as meta-analyses [5, 6, 7, 8, 9, 10, 11, 12, 13, 14, 15]. AIT further has been associated with reducing the risk of developing asthma [16] or other sensitizations [17]. However, a quite long therapy duration of at least 3 years is required to achieve long-term benefit [18, 19, 20, 21]. 

Unfortunately, treatment adherence has shown to be critical in clinical trials and real-life studies [22, 23, 24]. AIT can be administered subcutaneously (SCIT) or sublingually (SLIT). Both forms of administration are associated with aspects that could hinder adherence. SCIT, for instance, requires regular visits in the physician’s office, which may be associated with an increased time commitment, whereas successful therapy with SLIT requires daily intake [25, 26]. Lack of adherence reduces the effectiveness of AIT and further entails the use of limited resources without achieving the desired benefit [25, 27]. The aim of this review is to provide a targeted analysis of recent literature, to provide a picture of adherence in allergy immunotherapy in terms of extent and barriers. 

## Definition of adherence 

The World Health Organization defines adherence as the degree to which a patient’s behavior – e.g., taking medication – reflects the agreed recommendations of a healthcare provider [28, 29]. It is a process that encompasses three different phases: initiation (the event of taking the first dose of a medication), implementation (the extent to which the drug was used by the patient as prescribed during a specific period of active treatment), and persistence (the time period between initiation and discontinuation) [30]. More than one series of these stages are usually detected in routine practice [30]. Chronic diseases are widespread in Western countries [31]. For affected patients, adequate treatment of chronic illnesses is of great importance in order to prevent complications in their progression and to improve health outcomes and therefore the overall quality of life. Furthermore, the treatment of chronic diseases and their complications poses a relevant cost factor to the healthcare system [32]. 

Despite the fact that poor compliance reduces treatment benefit significantly and is associated with an increased risk of hospitalization and higher morbidity and mortality, compliance in chronically ill patients appears to be poor [29, 33]. Studies regarding this issue showed that only about half of the patients with chronic diseases take their medications as prescribed [34]. 

## Measurement of adherence in AIT 

Adherence research is generally compromised by the lack of standardization of adherence measures [35]. There is further no consensus on what percentage is considered good adherence and no gold standard established for quantifying adherence [33]. 

In general, measuring adherence requires determination of the number of doses actually taken by the patient. In the case of SLIT, this is technically difficult without the definite structures of a double-blind, placebo-controlled study. Some studies approached the problem by contacting the patients through unscheduled phone calls. However, questioning patients about their medication intake might not be very reliable, as patients are probably hesitant to admit incorrect or irregular use [6]. In addition, it should be noted that patients are aware of the study and fear consequences when reporting irregular intake; thus such studies might obtain an inherent bias [33]. 

## Reported adherence to AIT in the literature 

To date, several studies have been published on SCIT [6, 7, 36, 37, 38] and SLIT [22, 39, 40, 41, 42, 43, 44], showing overall poor adherence rates. 

Studies examining adherence to SCIT showed mixed results with adherence rates ranging from 23 to 89% [6, 12]. Studies examining adherence in SLIT showed rates varying from 64 to more than 95% [45, 46, 47]. 

There is still a controversial debate whether SCIT or SLIT might result in higher adherence. Some argue that SCIT might provide better adherence due to the regular visits at the physicians’ practice for the injections while others assume that SLIT results in better adherence because of its greater convenience [2, 23]. A German investigation based on a national prescription database comparing adherence rates with grass pollen SLIT to grass pollen SCIT over 3 years on a per-patient basis showed higher adherence to SLIT [23]. Furthermore, studies analyzing the literature on adherence in AIT found rates for SLIT to be better than SCIT [6, 12]. 

However, a survey in Italy reported an alarming rate of SLIT discontinuation, which was up to 90% at 3 years after prescription [48]. This particular study analyzed sales data from manufacturers and revealed an adherence rate to SLIT of only 15% at the 3^rd^ year. Such studies are of particular interest because they can circumvent the influence on patients that results from participation in a controlled trial. Real-life studies showed further great variations regarding adherence to AIT, from less than 50 to up to 90% [24, 26, 49, 50]. 

## Factors associated with adherence to AIT 

### Age 

With regard to age, differences in adherence could be demonstrated in various studies [2, 51, 52]. It is well known that genetic factors influence the development of allergic diseases. For this reason, parents of allergic children are often affected by allergic diseases themselves. We therefore assume that the experiences of disease burden might motivate parents to ensure that their children are adherent to AIT. Adherence among children generally depends heavily on their caregivers, and the motivation in parents to achieve a good therapeutic outcome for their children is likely to be high overall, regardless of whether the parents themselves are affected. 

Besides better adherence in children, there might be good adherence in elderly patients as well. A study conducted in the USA analyzed adherence in a group of veterans. Adherence rates were higher in patients older than 66 years than in young and middle-aged veterans. Considering that older patients are more likely to be retired, these findings suggest higher adherence in patients with less day-to-day commitment [51]. 

### Physicians 

Understanding the influence of physicians on patient adherence is of utmost importance as problems can be strategically addressed by healthcare providers themselves. 

Frequent visits and loss of working hours have been identified as reasons for patients to discontinue treatment. However, a trial by Vita et al. [22] actually found an improvement in adherence when performing regular therapy check-ups. A minimum of four visits per year was identified to ensure continuation of therapy. A further study by Egert-Schmidt et al. [2] confirmed these findings. The investigation showed better adherence in patients receiving perennial SCIT, who attended consultations more regularly. 

Moreover, the physician’s specialization seems to affect adherence. One study showed higher adherence in patients who were treated by general practitioners than in those treated by medical specialists [7]. In addition, compliance to AIT has also shown to be improved when prescribed and supervised by pediatricians [2]. Considering these results, it can be assumed that the trust relationship between doctor and patient is pivotal for adherence as it is greater when treated by general practitioners or pediatricians, and withdrawal rates can be reduced with regular visits. These results may be due to a greater overall extent of responsibility for the patient. In addition, patients may see their primary care physicians and pediatricians more regularly, apart from AIT, and therefore have more opportunities to address and improve problems related to treatment. Therefore, a standardized check-up schedule should be introduced, and the patient-doctor relation should be strengthened. 

### Efficacy 

AIT has been proven to be an effective treatment; however, its efficacy is limited by the compliance of the patient. Some studies showed good adherence in the beginning of therapy and rising drop-out rates in the 2^nd^ and 3^rd^ years of treatment [25, 52]. These findings probably reflect the greater motivation at the beginning of the therapy due to initial severity of symptoms. However, in cases where patients dropped out within the 1^st^ year of therapy, a lack of efficacy was often reported [37]. 

The 1^st^ year of therapy and the patients’ expectations can be assumed to have major impact on determining whether patients will adhere to therapy. For example, patients with milder disease courses may not have experienced a significant impact on their quality of life and may be less motivated to continue therapy in case of adverse effects or inconveniences. Other patients may have experienced sudden and significant results with other forms of therapy, such as nasal steroids and antihistamines, and therefore have unreasonably high expectations at the initiation of AIT [53]. 

### Inconvenience 

Studies by Musa et al. [25] and Hsu et al. [37]) found inconvenience to be a main reason for discontinuation of SCIT. Factors contributing to the inconvenience were the long duration of therapy as well as the high frequency of applications. Further, the application form itself and commuting to the place of administration were mentioned [25, 37]. 

Further reasons for discontinuation were adverse effects to the treatment [25, 54]. The latter was also stated to be the main reason for premature termination of a SLIT [25]. However, in this context it is interesting to note that new approaches to modify the AIT preparations have been investigated, which might be associated with a reduced occurrence of adverse advents [55, 56]. 

### Patients’ education 

A study by Sade et al. [57] in 2003 suggested that a significant number of patients receiving immunotherapy holds various misunderstandings and has a serious lack of knowledge about therapy duration, adverse effects, and their illness itself. However, a cross-sectional observational survey conducted in 2010 in Italy showed rather adequate levels of knowledge in patients. This investigation further showed the physicians to be the main source of information for patients [58]. 

Based on these results, it can be assumed that the patients’ knowledge gap is less grave than previously assumed. Nevertheless, there are numerous misunderstandings and a definite need for educating patients, which must be primarily addressed by the treating physician. 

Studies conducted in 2010 and 2020 demonstrated that investing time and resources in educating patients intensively about the received therapy might also improve adherence [59, 60]. A trial by Sanchez et al. [61] further suggested that patients should not only be provided with intensive information about their disease and the treatment process, but they should also be involved in the treatment decision. Their investigation showed that if patients could choose between SCIT and SLIT, adherence was significantly better [61]. 

As AIT is comparable to other long-term therapies, it might be useful to implement adherence measures, which have been found to be successful in research of other chronic diseases. Promising adherence interventions include standardized follow-up-schedules, improving communication and education via tools, and incorporating telecommunication technologies [62]. The use of new technologies appears particularly promising to improve treatment adherence in everyday life, as shown in a review article by Braido et al. [63]. 

### Reimbursement of AIT 

Financial aspects might also play a role in adherence. Vaswani et al. [64] study showed that inadequate reimbursement for allergen extract and allergy injections by health insurers is a common reason for non-adherence to SCIT [64]. A study investigating adherence to AIT in Turkey also found financial problems as main cause for discontinuation of therapy [65]. 

Another study demonstrated higher adherence in patients with a higher socioeconomic status [7]. Therefore, socioeconomic and work status may also be contributing factors to adherence to AIT. It is further important to consider regional differences depending on the healthcare system and the conditions for reimbursement of therapy costs. 

### Personalized medicine 

Previously, AIT was thought to take longer to achieve symptom reduction and to be less effective than other common symptom-relieving medications. Newer analyses implied however, that AIT in form of SCIT might be equally effective [66]. A decrease in allergic symptoms and the use of other medication can be observed within the 1^st^ year of therapy. However, a therapy duration of 3 years is necessary to achieve a lasting therapy effect. 

In order to achieve greater adherence, patients should be approached individually and included in the choice of therapy to improve acceptance. 

In general, SLIT might be more suitable for patients who work in time-consuming jobs, patients with needle phobia, or young children. SCIT on the other hand includes frequent check-ups and is preferred by patients who require greater support by their physician [67, 68, 69]. 

In order to improve adherence it must be considered that multiple factors are involved. As already emphasized in a previous review article, it therefore seems reasonable to apply different strategies that take into account the above-mentioned influencing factors [70]. 

### Monitoring adherence 

Various methods for measuring adherence have been applied while the reliability seems limited for each of them. 

Subjective measures such as questionnaires can lead to bias for several reasons. For example, the so-called Hawthorne effect can occur. In this case, an unconscious change in behavior occurs due to awareness of the survey. On the other hand, the additional attention that accompanies the examination can cause patients to respond according to a social desirability. In the context of this kind of examination, this means that respondents tend to answer questions in a way that is seen positively by others, in this case the doctor. 

As no method has been considered as gold standard for measuring adherence and no cut-off level for acceptable adherence rates has been set, further research is needed to provide adequate methods. Moreover, consensus on the application methodologies should be sought to achieve more standardization within the literature. 

### Real-life evaluations 

As mentioned, trials that investigate adherence usually contain a bias due to the patient’s awareness of the endpoint [6]. For this reason, data from RCTs may not adequately reflect adherence as participants are strictly followed and observed. Therefore, more consistency in data might be achieved via conducting real-life studies. The design of these studies is often retrospective in nature and relies on databases as primary sources [23, 52, 71]. This may be advantageous in this case because it allows a large number of patients and long periods of time to be observed without influencing the patients and therefore the outcome. Adherence rates in randomized controlled trials have been significantly higher which may be due to selecting and advising patients as well as effects due to the observation. Real-life studies bypass these effects and show results comparable to those in other chronic diseases with respect to adherence [72]. 

### Multi-national studies 

The literature on adherence to AIT is characterized by a strong heterogeneity which hinders deducing consequences for everyday clinical practice. In order to achieve large sample sizes in studies and to avoid bias due to region- and culture-specific influences, multi-national studies are needed [6, 73]. 

## Conclusion 

This review is intended to describe the data on adherence in AIT within the last 10 years, taking into account factors that influence adherence and highlight future perspectives and unmet needs. Adherence to AIT remains a pivotal point. 

To improve adherence, the following four points are of particular importance:

- Improving the patients’ knowledge about the treatment and their illness.
- Strengthening the partnership between doctors and patients.
- Providing reliable data in the form of real-life studies and multi-national studies with large numbers of participants.
- Standardization of measurement methods.

## Funding 

The manuscript was produced without financial help from a third party. 

## Conflict of interest 

Prof. Pfaar reports grants and personal fees from ALK-Abelló, grants and personal fees from Allergopharma, grants and personal fees from Stallergenes Greer, grants and personal fees from HAL Allergy Holding B.V./HAL Allergie GmbH, grants and personal fees from Bencard Allergie GmbH/Allergy Therapeutics, grants and personal fees from Lofarma, grants from Biomay, grants from Circassia, grants and personal fees from ASIT Biotech Tools S.A., grants and personal fees from Laboratorios LETI/LETI Pharma, personal fees from MEDA Pharma/MYLAN, grants and personal fees from Anergis S.A., personal fees from Mobile Chamber Experts (a GA2LEN Partner), personal fees from Indoor Biotechnologies, grants and personal fees from GlaxoSmithKline, personal fees from Astellas Pharma Global, personal fees from EUFOREA, personal fees from ROXALL Medizin, personal fees from Novartis, personal fees from Sanofi-Aventis and Sanofi-Genzyme, personal fees from Med Update Europe GmbH, personal fees from streamedup! GmbH, grants from Pohl-Boskamp, grants from Inmunotek S.L., personal fees from John Wiley and Sons, AS, personal fees from Paul-Martini-Stiftung (PMS), personal fees from Regeneron Pharmaceuticals Inc., personal fees from RG Aerztefortbildung, personal fees from Institut für Disease Management, personal fees from Springer GmbH, personal fees from AstraZeneca, personal fees from IQVIA Commercial, personal fees from Ingress Health, outside the submitted work; and member of EAACI Excom, member of ext. board of directors DGAKI; coordinator, main- or co-author of different position papers and guidelines in rhinology, allergology and allergen-immunotherapy. The rest of the authors declare that they have no relevant conflicts of interest. 
